# A Nutritional Approach for the Management of Deoxynivalenol (DON) Toxicity in the Gastrointestinal Tract of Growing Chickens

**DOI:** 10.3390/ijms9122505

**Published:** 2008-12-08

**Authors:** Wageha Awad, Khaled Ghareeb, Josef Böhm, Jürgen Zentek

**Affiliations:** 1 Institute of Nutrition, Department of Veterinary Public Health and Food Science, University of Veterinary Medicine, Veterinärplatz 1, A-1210 Vienna, Austria; 2 Department of animal Behaviour and Management, Faculty of Veterinary Medicine, South Valley University Qena, Egypt; 3 Institute of Animal Nutrition, Department of Veterinary Medicine, Freie University of Berlin, Brümmerstr. 34, D-14195 Berlin, Germany

**Keywords:** Deoxynivalenol, inulin, electrophysiology, ussing chambers, poultry

## Abstract

It has been shown that DON has negative effects on the active transport of some nutrients in the small intestine of chickens. The plausible interactions between food contaminants and natural components could be high. The present study investigated the effects of DON on the presence or absence of dietary inulin on the electrophysiological response of the gut to glucose. Ussing chamber studies were conducted with isolated jejunal epithelia at the age of 35 days. Electrophysiology of the epithelia was recorded and the changes of the short-circuit current (Isc) were determined. The addition of d-glucose on the luminal side of the isolated mucosa increased (P < 0.05) the Isc in the control group and inulin supplemented group. The oligosaccharides did not increase glucose absorption in young healthy chickens compared with the controls. In the second experiment, after preincubation of tissues with DON, the addition of glucose did not increase the Isc in jejunum and colon in the control group (P > 0.05). However, in the dietary inulin supplemented group in both jejunum and colon, the addition of glucose after preincubation of tissues with DON increased the Isc, suggesting that the dietary inulin supplementation of the broilers regulated and improved the glucose absorption in the presence of DON and kept it at normal levels.

## 1. Introduction

One of the major concerns for the poultry industry is the threat of mycotoxins associated with poultry feedstuffs. Mycotoxins were ranked as the most important chronic dietary risk factors, higher than synthetic contaminants, plant toxins, or pesticide residues [1–2]. Oldenburg *et al*. [3] emphasized that a certain risk remains of mycotoxin production by several *Fusarium* species despite application of a number of preventive steps in plant production, because of the high influence of the weather conditions at the time of cereal flowering. DON altered small intestinal morphology, especially in the duodenum and jejunum, as evidenced by shorter and thinner villi [4]. It has been shown that DON decreased glucose and amino acid absorption in the chicken small intestine after dietary exposure and *in vitro* [5–8]. DON is the major trichothecene in feedstuffs in Central Europe and worldwide. Therefore, strategies have to be developed or applied in order to cope with cereal batches which are contaminated with DON. Complete elimination of any natural toxin from foods is an unattainable objective.

Nutritional science has been expanding the knowledge of how feed additives influence consumers in relation to specific health parameters. Foods are no longer appreciated only in terms of their taste and immediate nutritional needs, but also in terms of their ability to provide specific health benefits. Prebiotics can be classified as non-digestible oligosaccharides that cannot be hydrolysed by enzymes of endogenous origin. As a consequence, prebiotics are quantitatively available as substrates for the gastrointestinal microflora [9] and affect the host beneficially by selectively stimulating the growth and/or metabolic activity of one or more naturally present bacterial species in the intestine [10].

Inulin, a prebiotic extracted from chicory (*Cichorium intybus*), contains both oligosaccharide components and polysaccharides. Rehman *et al*. [11] found that inulin stimulates the metabolic activity of the intestinal microflora and also increases villus height and crypt depth of jejunal mucosa of broilers fed with an inulin supplemented diet. In addition, they found a lower transmural tissue resistance in jejunal sheets from broilers fed 1% inulin compared to controls. Suzuki and Hara [12] reported that application of different non-digestible oligosaccharides can increase the paracellular permeability of Caco-2 cell monolayer mounted in Ussing chamber. *In vitro* application of fructooligosaccharides (FOS) and inulin to rat intestinal sheets mounted in Ussing chamber increased paracellular permeability resulting in an increased conductance of calcium across the intestinal mucosa [13]. Many of the physiological effects associated with inulin consumption are directly linked to its selective promotion of specific strains of gut microorganisms, particularly bifidobacteria and lactobacilli [14]. Many studies found that inulin aid in the control of glucose absorption and maintaining the glucose concentration at normal levels [15–19]. Generally, inulin may have a significant impact on the management of mycotoxin contamination.

The interaction between DON and prefeeding prebiotic (inulin) and the effect of inulin in the presence of DON on the electrophysiological parameters has not yet been studied. Therefore, an *in vitro* study was conducted to evaluate the effect of inulin on the electrophysiological parameters in the presence and absence of the mycotoxin deoxynivalenol in the chicken gut.

## 2. Materials and Methods

### 2.1. Birds and feeding

Forty day-old broilers of a commercial strain (Ross 308), obtained from a commercial hatchery, were randomly divided into two groups. The birds were housed in four wire-bottomed pens for the 35 day experimental period. The temperature started at 33 °C (from day 0 to day 3) and was gradually reduced (2–3 °C/week). Chicks were maintained on an 18 hours constant light schedule until the end of the experiment. Chicks in the control group were fed a basal diet, which was mainly comprised of maize and soya (Table 1). The second group was fed the basal diet with 1.0% inulin (Orafti Active Food Ingredients, Tienen, Belgium). Feed and water were provided *ad libitum*.

### 2.2. Tissue preparation

Tissue preparation, incubation in Ussing chambers and electrophysiological recordings were performed as described earlier [5]. Birds were slaughtered by stunning and bleeding. Immediately after exsanguinations, segments were taken from the mid-jejunum and colon. Segments from colon were taken to be representative for the effect of inulin in the large intestine and for comparing it with the small intestine. Additionally, in our previous study [20] we found that DON decreased the glucose transport of all intestinal regions. The intestine was rinsed with ice-cold buffer and transported to the laboratory in ice-cold incubation buffer oxygenated with carbogen (95% O_2_/5% CO_2_). The intestinal segment was opened along the mesenteric border and washed free of intestinal contents with buffer solution at 4 °C. Segments were kept in cold buffer solutions being continuously gassed with carbogen until mounted in Ussing chambers.

### 2.3. Isolated intestinal tissues in using chambers

After the preparation of the intestinal sheets, the tissue was mounted in modified Ussing chambers with an active area of 1 cm^2^. The Ussing chamber allows the measurement of actively transported ions as well as the permeability of the tissues, two parameters relevant for the evaluation of gut health. The serosal and mucosal surfaces of the tissues were bathed in 5 mL Ringer solution with the following composition (mmol/L): CaCl_2_, 1.2; MgCl_2_, 1.2; Na_2_HPO_4_, 2.4; NaH_2_PO_4_, 0.4; NaHCO_3_, 25; KCl, 5; NaCl, 115; mannitol, 20. The buffer solutions were adjusted to pH 7.4 at 38 °C with NaOH or HCl. Continuous oxygenation provided by recirculation of the incubation solutions by means of a gas lift. After a tissue equilibration period of 20 min, the basal measurements of current (Isc), and tissue resistance (Rt) were recorded. When the tissue was stabilized, the 5 mL of Ringer solution on the luminal side was replaced by 5 mL of glucose buffer. The electrical response to glucose was measured as the peak response obtained approximately 1 min after the addition of the substrate. Moreover, 50 μg DON/5 mL was added to the luminal side after the addition of d-glucose to investigate the effect of DON on Na^+^-d-glucose co-transporters. Further experiments were performed to investigate the mechanisms of action of DON. The tissues were incubated with 5 mL of Ringer solution on the luminal side containing 50 μg DON/5 mL of Ringer solution for 1 hr. Thereafter, d-glucose (5 mmol/L) was added to the luminal side after preincubation of the tissues with DON.

### 2.4. Statistics

Statistical analyses were conducted with the SPSS. The Kolmogorov Smirnov test was used to test the normal distribution of the data before statistical analysis was performed. Results are expressed as means and the SEM was pooled. To investigate the effect of DON and inulin on the Isc, and Rt, a paired t test was used between each two pairs of repetitions within the same segment. Independent samples t-test was used for group comparisons. Probability values (P) of 0.05 or less were considered significant.

## 3. Results

The dietary inclusion of inulin (1%) slightly improved the growth performance in broilers, whereas the mean body weight over the course of the experiment was numerically higher. The villus height and crypt depth of the jejunal mucosa were increased (P < 0.05) for birds fed the diet supplemented with inulin compared to the control group (data not shown).

### 3.1. Effect of DON addition after glucose addition on tissue resistance and short-circuit current

The addition of 5 mmol/L d-glucose on the luminal side of both jejunum and colon increased the Isc (P = 0.09) in the control and inulin-supplemented groups, while this parameter decreased (P = 0.019) after addition of 10 μg DON/mL to the mucosal solution within 30 min (Table 2). The tissue resistance (Rt) in the control and inulin birds was similar, and no significant difference (P > 0.05) was noted between control and the dietary inulin-supplemented groups. However, the Rt was higher (P < 0.05) in the jejunum exposed to DON in control group (423 Ω·cm^2^) compared with the basal values (342 Ω·cm^2^) and the values after addition of glucose (360 Ω·cm^2^). While in inulin-supplemented groups the Rt remained unchanged after addition of either glucose or DON in both jejunum (336, 369 Ω·cm^2^ respectively) and colon (123, 127 Ω·cm^2^ respectively) and it was numerically lower compared with the controls (Table 3).

### 3.2. Effect of D-glucose addition on tissue resistance and short-circuit current after preincubation of the tissues with DON

The Isc was not increased (P > 0.05) in jejunum and colon in the control group (Table 4) by the addition of glucose after preincubation of tissues with DON, suggesting that DON inhibited the Na^+^ d-glucose co-transport. However, in the dietary inulin supplemented group in both jejunum and colon, the addition of glucose after preincubation of tissues with DON increased the Isc (P ≤ 0.05), suggesting that the dietary inulin supplementation improved the glucose transport in the presence of deoxynivalenol. The addition of d-glucose on the mucosal side after preincubation of the tissues with DON for 1 hr by paired t test increased the Rt (P < 0.05) in jejunum in control group (374 Ω·cm^2^) compared with basal Rt value (320 Ω·cm^2^) (Table 5). However, the resistance of colon and jejunal tissues remained unaffected by the dietary inulin supplementation and there was no significant difference (P > 0.05) between the control and inulin birds.

## 4. Discussion

The intestinal microbiota plays a vital role in the normal nutritional, physiological, immunological, and protective functions of the host animals [21]. The composition and metabolic activity of the intestinal microbiota can be influenced by the diet [22]. There is a growing interest in the use of a variety of oligosaccharides as prebiotics to promote animal health by altering the intestinal microbial community. A prebiotic is a selectively fermented ingredient that allows specific changes, both in the composition and/or activity in the gastrointestinal microbiota and that is not digested by the host digestive enzymes [23]. In theory, prebiotics selectively stimulate the growth of endogenous lactic acid bacteria and *Bifidobacteria* [14].

Supplementing the diet with 1.0% oligofructose or 1.3% inulin increased pancreatic amylase, serum glucose concentration, and abilities to absorb the higher amounts of digested protein in the jejunum of laying hens, but without an increase in glucose transport at normal condition [24]. Many studies showed that villus height of the small intestine was increased in broilers supplemented with fermentable carbohydrates like fructooligosaccharides (FOS) [25–26] and inulin [11]. It has been shown that an increased villus height is paralleled by an increased digestive and absorptive function of the intestine due to increased absorptive surface area, expression of brush border enzymes and nutrient transport systems [27]. Suzuki and Hara [12] reported that application of different non-digestible saccharides can increase paracellular permeability. Studies in humans demonstrated that inulin and other dietary fiber improved blood glucose control and reduced the number of hypoglycaemic events in diabetic patients [16, 18].

The result of present study reveals non-significant difference in basal Isc in both dietary groups. This is in accordance with findings of Nancy *et al*. [28], who could not find differences in basal Isc for jejunum and colon of pigs supplemented with different oligosaccharides compared to control group. The glucose addition on the luminal side of the jejunum and colon increased the Isc in the control and dietary inulin-supplemented groups, but there was no difference between the control and the dietary inulin-supplemented groups. The results are in agreement with Rehman *et al*. [11] who showed that there was no difference in glucose absorption between inulin supplemented group and controls. The present study reveals that the tissue resistance of jejunal and colon tissues were similar in the inulin-supplemented group and control group. This is in agreement with Nancy *et al*. [28] who noted that the resistance of colon and jejunal tissues remained unaffected by the dietary supplementation with FOS. However, the Rt was higher in the jejunum exposed to DON in control group compared with the basal values. These findings are in agreement with Awad *et al*. [6–7], who found that the Rt was higher in the tissues exposed to DON.

In the second experiment, the Isc was not increased in the jejunum and colon in the control group by the addition of glucose after preincubation of tissues with DON, demonstrate that DON inhibited the glucose transport. The results of this study are in agreement with our previous findings [5–8, 20] that DON has a specific inhibitory effect on glucose absorption mediated by SGLT-1. However, in the dietary inulin supplemented group in both jejunum and colon, the addition of glucose after preincubation of tissues with DON increased the Isc. As far as we know, no information exists regarding the influence of inulin in the presence of DON on the electrical variables of the intestine of broilers. In the present study, inulin appeared to improve the glucose transport in the presence of DON in commercial broilers. Many of the physiological effects associated with inulin consumption are directly linked to its selective promotion of specific strains of gut microorganisms, particularly bifidobacteria and lactobacilli. The bifidobacteria and lactobacilli produce short chain fatty acids (SCFA) and lactic acid and a range of other antimicrobial compounds. Trappenden and McBurney [29] found that an increase in SCFA contributed to elevated serum GLUT2 (a glucose transporter) and serum glucagon-like peptide- (GLP-2) and proglucagon mRNA in the ileal region of rat intestine. The present study indicated that inulin supplementation improved the glucose absorption in the presence of DON. This may be linked to increase passive absorption of glucose and facilitate its transport in the gastrointestinal tract [30]. This may offer the host protection against the negative effects of DON on intestinal glucose absorption.

## 5. Conclusions

In conclusion, from this study we can conclude that the use of inulin offers a promising approach to restore microbial communities and to support barrier function of the gut epithelia by their prebiotic action. This may offer the host protection against the negative effects of DON on intestinal glucose absorption. However, further studies with other feeding combinations of inulin and DON need to be done to extend these findings.

## Figures and Tables

**Table 1.** Ingredient percentages and analysis of basal diet.

Ingredients	Composition
Maize	50.50
Soya meal	30.00
Wheat	10.00
Potato Protein	3.00
Soya oil	1.40
Sodium chloride	0.10
Vitamin-Mineral Mixture^1^	5.00
Analysed composition (DM)	
Dry matter (%)	87.9
Ash (%)	6.6
Crude fiber (%)	1.5
Crude protein (%)	24.9
Crude fat (%)	3.5
Calculated ME (kcal/kg)	2,820
Calcium (%)	1.08
Phosphorous (%)	0.87
Magnesium (%)	0.26
Sodium (%)	0.33
Potassium (%)	1.27

1 BR 5 Universal Vetmed, Biomin GmbH, Herzogenburg, Austria. Each kg contains calcium 196 g, phosphorous 64 g, sodium 30 g, magnesium 6 g, copper 400 mg, zinc 1200 mg, iron 2000 mg, manganese 1200 mg, cobalt 20 mg, iodine 40 mg, selenium 8 mg, vitamin A 200,000 IU, vitamin D_3_ 80,000 IU., vitamin E 1600 mg, vitamin K_3_ 34 mg, vitamin C 1300 mg, vitamin B_1_ 35 mg, vitamin B_2_ 135 mg, vitamin B_6_ 100 mg, vitamin B_12_ 670 mcg, nicotinic acid 1340 mg, calcium pantothenic acid 235 mg, choline chloride 8400 mg, folic acid 34 mg, biotin 3350 μg, methionine 30g.

**Table 2.** The short-circuit current (Isc) in isolated jejunal and colonic mucosa of broilers after addition of glucose and DON.

Parameters /Organs	Groups		SEM	P value
	Control	Inulin		
**1- Jejunum**				
Basal Isc (μA/cm^2^)	32^b^	7^b^	8	0.203
Isc after glucose addition (μA/cm^2^)	53^a^	33^a^	7	0.135
Isc after DON addition (μA/cm^2^)	3^b^	3^b^	7	0.974
**2- Colon**				
Basal Isc (μA/cm^2^)	−32^b^	−5^b^	17	0.234
Isc after glucose addition (μA/cm^2^)	−2^a^	30^a^	11	0.241
Isc after DON addition (μA/cm^2^)	−63^b^	−5^b*^	20	0.083

Different letters indicate significant differences within a column (P ≤ 0.05, Paired t test). The results are reported as means, SEM (pooled), (n= 5/treatment).

**Table 3.** The electrical tissue resistance (Rt) in isolated jejunal and colonic mucosa of broilers after addition of glucose and DON.

Parameters /Organs	Groups		SEM	P value
	Control	Inulin		
**1- Jejunum**				
Basal Rt (Ohm.cm^2^)	342^b^	330	36	0.477
Rt after glucose addition (Ohm.cm^2^)	360^b^	336	38	0.186
Rt after DON addition (Ohm.cm^2^)	423^a*^	369	47	0.070
**2- Colon**				
Basal Rt (Ohm.cm^2^)	160	148	6	0.275
Rt after glucose addition (Ohm.cm^2^)	144	123	8	0.148
Rt after DON addition (Ohm.cm^2^)	139	127	6	0.284

Within the same column means with different superscripts are significantly different (P ≤ 0.05, Paired t test), The results are reported as means, SEM (pooled), (n= 5/treatment)

**Table 4.** Short-circuit current (Isc) across the isolated jejunal and colonic mucosa of broilers after preincubation of the tissues with DON and after glucose addition.

Parameters /tissue	Groups		SEM	P value
	Control	Inulin		
**Jejunum**				
Basal Isc with DON (μA/cm^2^)	−7	3^b^	6	0.421
Isc after glucose addition (μA/cm^2^)	−7	6^a^	6	0.233
**Colon**				
Basal Isc with DON (μA/cm^2^)	14	−40^b^	25	0.318
Isc after glucose addition (μA/cm^2^)	−3	69^a^	34	0.330

Within the same column means with different superscripts are significantly different (P ≤ 0.05, Paired t test), The results are reported as means, SEM (pooled), (n= 5/treatment).

**Table 5.** The electrical tissue resistance (Rt) across the isolated jejunal and colonic mucosa of broilers after preincubation of the tissues with DON and after glucose addition.

Parameters /tissue	Groups		SEM	P value
	Control	Inulin		
**Jejunum**				
Basal Rt with DON (Ω·cm^2^)	320^b^	378	33	0.088
Rt after glucose addition (Ω cm^2^)	374^a^	391	32	0.212
**Colon**				
Basal Rt with DON (Ω cm^2^)	166	145	11	0.380
Rt after glucose addition (Ω cm^2^)	135	128	8	0.688

Different letters indicate significant differences within a column (P ≤ 0.05, Paired t test), The results are reported as means, SEM (pooled), (n= 5/treatment).

## References

[1] Kuiper GG, Lemmen JG, Carlsson B, Corton JC, Safe SH, Saag PT, Burg B, Gustafsson JA (1998). Interaction of estrogenic chemicals and phytoestrogens with estrogen receptor beta. Endrocrinology.

[2] Bennett JW, Klich M (2003). Mycotoxins. Clin. Microbiol. Rev.

[3] Oldenburg E, Valenta H, Sator CH, Dänicke S, Oldenburg E (2000). Risikoabschätzung und Vermeidungsstrategien bei der Futtermittelerzeugung. Risikofaktoren für die Fusariumtoxinbildung und Vermeidungsstrategien bei der Futtermittelerzeugung und Fütterung.

[4] Awad WA, Böhm J, Razzazi-Fazeli E, Ghareeb K, Zentek J (2006). Effect of addition of a probiotic microorganism to broiler diets contaminated with deoxynivalenol on performance and histological alterations of intestinal villi of broiler chickens. Poult. Sci.

[5] Awad WA, Böhm J, Razzazi-Fazeli E, Hulan HW, Zentek J (2004). Effects of deoxynivalenol on general performance and electrophysiological properties of intestinal mucosa of broiler chickens. Poult. Sci.

[6] Awad WA, Böhm J, Razzazi-Fazeli E, Zentek J (2005a). *In Vitro* Effects of deoxynivalenol on electrical properties of intestinal mucosa of laying hens. Poult. Sci.

[7] Awad WA, Rehman H, Böhm J, Razzazi-Fazeli E, Zentek J (2005b). Effects of luminal deoxyn ivalenol and L-proline on electrophysiological parameters in the jejunums of laying hens. Poult. Sci.

[8] Awad WA, Aschenbach JR, Setyabudi FMCS, Razzazi-Fazeli E, Böhm J, Zentek J (2007a). *In vitro* effects of deoxynivalenol on small intestinal D-glucose uptake and absorption of deoxynivalenol across the isolated jejunal epithelium of laying hens. Poult. Sci.

[9] Roberfroid MB, Loo JAE, Gibson GR (1998). The bifidogenic nature of chicory inulin and its hydrolysis products. J. Nutr.

[10] Young RJ, Whitney DB, Hanner TL, Antonson DL, Lupo JV, Vanderhoof JA (1998). Preventing of antibiotic-associated diarrhea utilizing Lactobacillus GG. Gastroenterol. Int.

[11] Rehman H, Rosenkranz C, Böhm J, Zentek J (2007). Effects of inulin on histomorphology and electrophysiological indices of the jejunal mucosa in broilers. Poult. Sci.

[12] Suzuki T, Hara H (2004). Various nondigestible saccharides open a paracellular calcium transport pathway with the induction of intracellular calcium signalling in human intestinal Caco-2 cells. J. Nutr.

[13] Raschka L (2005). Mechanisms underlying the effects of inulin-type fructans on the intestinal calcium absorption.

[14] Gibson GR, Beatty ER, Wang X, Cummings JH (1995). Selective stimulation of bifidobacteria in the human colon by oligofructose and inulin. Gastroenterology.

[15] Fuessl HS, Williams G, Adrian TE, Bloom SR (1987). Guar sprinkled in food: effect on glycaemic control, plasma lipids and gut hormones in non-insulin dependent diabetic patients. Diabet. Med.

[16] Buyken AE, Toeller M, Heitkamp G, Vitelli F, Stehle P, Scherbaum WA, Fuller JH (1998). Relation of fiber intake to HbA1c and the prevalence of severe ketoacidosis and severe hypoglycemia. Diabetologia.

[17] Chandalia M, Garg A, Lutjohann D, Von Bergmann K, Grundy SM, Brinkley LJ (2000). Beneficial effects of high dietary fiber intake in patients with type 2 diabetes mellitus. N. Engl. J. Med.

[18] Giacco R, Parillo M, Rivellese AA, Lasorella G, Giacco A, D’Episcopo L, Riccardi G (2000). Long-term dietary treatment with increased amounts of fiber-rich low-glycemic index natural foods improves blood glucose control and reduces the number of hypoglycaemic events in type 1 diabetic patients. Diabetes Care.

[19] Vuksan V, Jenkins DJA, Spadafora P, Sievenpiper JL, Owen R, Vidgen E, Brighenti F, Josse R, Leiter LA, Bruce-Thompson C (1999). Konjac-mannan. (glucomannan) improves glycemia and other associated risk factors for coronary heart disease in type 2 diabetes: A randomized controlled metabolic trial. Diabetes Care.

[20] Awad WA, Razzazi-Fazeli E, Böhm J, Zentek J (2007b). Influence of deoxynivalenol on the D-glucose transport across the isolated epithelium of different intestinal segments of laying hens. J. Anim. Nutr. Anim. Physiol.

[21] Vispo C, Karasov WH, Mackie RJ, White BA, Issacson RE (1997). Interaction of Avian Gut Microbes and their Host: An Exclusive Symbiosis. Gastrointestinal Microbiology 1. Gastrointestinal Microbes and Host Interactions.

[22] Netherwood T, Gilbert HJ, Parker DS, O’Donnell AG (1999). Probiotics shown to change bacterial community structure in the avian gastrointestinal tract. Appl. Environ. Microbiol.

[23] Gibson GR, Probert HM, Van Loo J, Rastall RA, Roberfroid MB (2004). Dietary modulation of the human colonic microbiota: Updating the concept of prebiotics. Nutr. Res. Rev.

[24] Chen YC, Nakthong C, Chen TC, Buddington RK (2005). The influence of a dietary beta-fructan supplement on digestive functions, serum glucose, and yolk lipid content of laying hens. Int. J. Poult. Sci.

[25] Sonmez NW, Eren V (1999). Effects of supplementation of zinc bacitracin, mannan-oligosaccharides and probiotic into the broiler feeds on morphology of the small intestine. Vet. Fak. Derg. Uludag Univ.

[26] Xu ZR, Hu CH, Xia MS, Zhan XA, Wang MQ (2003). Effects of dietary fructooligosaccharide on digestive enzyme activities, intestinal microflora and morphology of male broilers. Poult. Sci.

[27] Pluske JR, Tompson MJ, Atwood CS, Bird PH, Williams IH, Hartmann PE (1996). Maintenance of villus height and crypt depth, and enhancement of disaccharide digestion and monosaccharide absorption, in piglets fed on cow’s whole milk after weaning. Br. J. Nutr.

[28] Correa-Matos NJ, Donovan SM, Isaacson RE, Gaskins HR, White BA, Tappenden KA (2003). Fermentable fiber reduces recovery time and improves intestinal function in piglets following *Salmonella typhimurium* infection. J. Nutr.

[29] Tappenden KA, McBurney MI (1998). Systemic short-chain fatty acids rapidly alter gastrointestinal structure, function, and expression of early response genes. Dig. Dis. Sci.

[30] Chichlowski M, Croom WJ, Froetschel MA, Koci MD, McBride BM, Qiu R, Daniel LR (2006). Effect of PrimaLac, direct fed microbial, on ileal absorption, energy expenditure and intestinal microbial fermentation. Poult. Sci.

